# Supplementary material for: Are carbon emissions trading and green financial instruments synergistic? -Comprehensive quantitative research based on content analysis
Source: PLoS One. 2024 Mar 7;19(3):e0298601. doi: 10.1371/journal.pone.0298601 (PMC10919601; doi:10.1371/journal.pone.0298601)
Supplement: S1 Data — (ZIP) [file pone.0298601.s001.zip › Data/地级市Z_F工作报告.zip!/╔╜╬≈/╜·╓╨╩╨/└┤╫╘╣1⁄2╓┌║┼í╛┬φ┐╦╩2╛▌═°í┐_2010.docx]

各位代表：

本届政府2005年6月就职，任期即将结束。现在，我代表市人民政府向大会作报告，请予审议，并请政协委员和其他列席人员提出意见。

一、过去五年工作回顾

五年来，在省委、省政府和市委的坚强领导下，在市人大、市政协的有力监督和支持下，我们以“三个代表”重要思想为指导，紧扣“加快科学发展、构建和谐晋中”主题，团结带领全市人民，抢抓机遇，负重拼搏，全面实施“十一五”战略部署，市二届人大提出的目标任务全部完成，市人民政府历年向全市人民承诺的实事全部兑现！

五年来，主要取得了以下几方面的成绩：

（一）坚定不移实施赶超战略，综合实力再攀新高。

 经济进入加速成长阶段。全市地区生产总值由281.7亿元增加到636.8亿元，年均增长12％。人均地区生产总值接近3000美元。规模以上工业增加值由97.3亿元增加到276.2亿元，年均增长17.2%，是晋中历史上工业经济发展最快的时期之一。

 增长后劲不断加大。五年全社会固定资产投资累计完成1363亿元，是上个五年的4倍，年均增长26.6%。外贸进出口总额累计完成22.1亿美元，是上个五年的2倍。社会消费品零售总额由101.8亿元增加到249亿元，年均增长19.6%。

 财政实力全面提高。全市财政收入突破百亿大关，由35.4亿元增加到125.5亿元，年均增长28.8%。一般预算收入由13亿元增加到57.8亿元，年均增长34.8%。灵石、介休财政收入进入全省十强。东部山区和平川地区发展差距明显缩小。

 人民生活更加殷实。城乡居民储蓄存款余额由295亿元增加到706亿元，年均增长19.1％。城镇居民人均可支配收入由6943元增加到14628元，超过全省平均水平。农民人均纯收入由2949元增加到5194元，增幅居全省前列。城乡收入比为2.82，低于全省平均水平。五年减少贫困人口7万人。2009年，居民消费价格总水平下降0.1%。

 经过五年的努力，全市主要经济指标在全省的位次实现前移！

（二）深入推进经济结构战略性调整，发展方式明显转变。

 工业新型化进程加快。五年新增规模以上工业企业140户，规模以上工业增加值和高新技术企业增加值占GDP比重分别达到43.4%和3.5%。工业园区产值比重达到35%。循环经济规模扩大。工业新型化水平由29.7%提高到50.8%。2009年，强力推进煤炭资源整合、煤矿兼并重组，全市煤炭核准产能增加到1亿吨以上，平均单井产能提高到87万吨以上，回采率达到65%以上，煤炭对工业增长的贡献率达到111.5%，煤炭工业进入全新发展阶段。

 现代服务业拉开布局。“3+7”战略全面实施，以物流、旅游、金融、房地产等为重点的现代服务业发展态势强劲，服务业增加值占GDP比重达到38.9%。山西能源工业国际分销物流港等9个重大物流项目进展顺利。2009年，全市旅游总收入90亿元，是2004年的6.8倍。

农业产业化水平不断提升。启动了现代农业示范区建设，开工67个重点项目。发展3户国家级农业产业化龙头企业。农产品商品率、加工率分别达到95%和35%。农产品“三品”认证482个。发展农民专业合作社4254个。全市粮食生产连年丰收，蔬菜和肉蛋奶总产稳居全省前列。

 项目带动效应进一步显现。五年累计实施5000万元以上项目520个，榆次国电一期2×30万千瓦电厂、介休三佳6万吨有机硅等一批调产项目建成投产。2009年，共争取四批扩大内需中央投资项目389项，完成投资12.3亿元，基本完工175项。全年施工项目1553个，完成投资395.9亿元，投产项目420个，项目总量之大、开工之多、推进之快为历年之最。

 节能减排和环境保护取得重大进展。大力实施蓝天碧水工程、汾河清水复流综合治理工程和造林绿化工程，推行“双百”企业节能行动计划。万元GDP综合能耗累计下降21.98%，万元工业增加值能耗累计下降30.6%。二氧化硫、化学需氧量分别削减1.67万吨、0.32万吨。林木绿化率由21.7%提高到29.2%。市城区空气质量二级以上天数由168天增加到348天。

（三）从战略高度谋划和推进城乡建设，城市承载能力显著提升。

 城市框架逐步拉开。五年全市实施重点规划230项，开工市政重点工程613项，完成投资106.1亿元。建城区面积扩大4.4平方公里，北部新城建设顺利启动。县城和小城镇建设进程加快，城镇化水平达到43.5%。仅2009年，市城区竣工市政工程11类38项，完成投资9.53亿元，创历年新高。

 城市功能日益完善。深入开展“四城联创”活动，污水处理率、垃圾处理率、燃气普及率、集中供热普及率分别增加54个、22个、25个、33个百分点。市城区新增绿化面积677.1万平方米，人均公园绿地达到10平方米。新增管网446.7公里、热电联供面积300万平方米。市城区和11个县（区、市）通过省级卫生城市验收。

 基础设施建设整体突破。路网框架进一步完善，太长高速建成通车，汾邢高速全线启动，龙城高速奠基开工，东纵高速铺开前期，太中银、大西客专晋中段全面开工。五年新增公路2584公里，村村通里程比上个五年增加4倍，通达率达到99.3%。全面实施兴水战略，寿阳松塔等6个骨干水源工程建设加快，23座中小型病险水库完成加固，建成农村饮水安全工程1293处，新增和改善节水灌溉面积50万亩。电网结构更加合理，五年累计完成投资34.5亿元，新增35千伏及以上变电容量5012兆伏安，新增发电装机容量88.8万千瓦。

 新农村建设有序推进。强农惠农政策全面落实。各级财政累计投入新农村建设资金6.6亿元。“创三优”、“四化四改”工程全面实施，500个村达到“十有十个一”标准，建成园林村823个。

（四）大力推进改革开放，加快建设一流发展环境。

 各项改革不断深化。国企改革步伐加快，五年完成96户国有企业改革任务，改制面达到92.3%。2009年，锦纶、晋华、轻机3户国有企业宣告破产，进入法律程序。政府机构改革、文化体制改革、公共财政体制改革、农村综合改革等富有成效。

 对外开放进一步扩大。主动融入太原都市圈，积极推进晋中太原同城发展。多形式持续开展招商引资，五年引进项目412个，实际利用外资3.9亿美元、市外资金375亿元。

发展环境全面优化。深入开展“七个一流”创建活动。行政许可审批事项由414项压减至276项，审批时限平均缩短56.2%。建立了市县乡村四级政务服务网络、网上行政审批、电子监察和移动办公系统。全面推行“宁静生产日”、重大项目审批政府代理等制度。金融生态环境建设取得重大进展，引进了浦发银行、兴业银行、华夏银行，组建了商业银行、邮储银行，筹建榆次、左权村镇银行。企业上市工作积极推进。各类保险公司达到21家，大同证券落户晋中。中小企业信用担保体系初步建立，13个政策性担保公司注册资本金达到4.7亿元。21亿元民间资本通过33户商业性小额贷款组织进入信贷领域。农村小额人身保险全国领先。仅2009年，市财政向市中小企业信用担保公司注资3100万元，各类金融机构新增贷款79.1亿元，同比增长126.8%。“五缓四降一补”政策共为企业减负6180万元。

（五）始终把改善民生作为工作的出发点和落脚点，和谐晋中建设扎实推进。

 民生投入不断加大。五年财政用于民生领域支出248.7亿元，年均增长33.6%。其中，2009年76.3亿元，占一般预算支出的73.9%。城乡就业更加充分。建立了“九个一批”就业模式，五年新增城镇就业20.2万人，转移农村劳动力39.4万人。2009年，城镇登记失业率1.97%。

 社会保障体系进一步健全。各类保险参保人数、保费征缴额逐年提高。2009年，职工失业和工伤保险实现市级统筹。经济适用房、廉租房建设步伐加快，五年城镇居民和农村居民人均住房面积分别增加5.6平方米和3.9平方米。社会救助更加有力。城乡低保实现应保尽保。

 社会事业全面发展。“五大惠民工程”和“五个全覆盖工程”有效实施。科技工作和自主创新能力不断强化。全市研究与试验发展经费支出占GDP比重逐年提高，每10万人申请专利15.3件。教育事业优先发展。农村义务教育“两免一补”实现全覆盖；学校布局结构优化，义务教育学校调减为1182所，普通高中整合为34所，改造中小学校危房28.5万平方米；组建了晋中职教集团，普职高招生比例保持在1：0.74；2009年高中阶段普及率、高考达线人数、万人达线率、职业教育招生数居全省第一。城乡公共卫生和基本医疗服务体系初步建立。市县乡医疗卫生机构改扩建面达到90%，村卫生室实现全覆盖；县乡村三级医疗机构达标率达到95%；新建55个城市社区卫生服务中心和服务站；新农合参合率和基金使用率分别达到93%和85%。“文化晋中”建设深入推进。文化产业增加值占GDP比重4.8%，超过全国、全省平均水平；晋中文化生态保护区项目通过国家评审；93项非物质文化遗产列入国家、省、市保护名录；涌现出电视剧《乔家大院》等一批文艺精品。竞技体育、群众体育全面发展，人均公共体育场地面积达到1.5平方米。食品药品放心工程深入实施。人口与计生、外事、侨务、气象、防震、档案、文物、地方志、民族、宗教、广电、新闻出版、人民防空、国防后备力量建设等工作取得新成绩，老龄、妇女、残疾人、关心下一代等事业都有新进步。

安全生产形势保持平稳。高度重视安全生产，坚持“双逐月”等一系列安全监管制度，严格责任、强化落实。2009年，全市各类生产事故发生起数、死亡人数同比下降18.2%、8.4%。

（六）民主法制继续加强，精神文明不断发展。

 民主法制建设深入推进。依法治市全面实施。各级政府严格依法行政，自觉接受人大、政协及社会各界的监督。五年市政府共办结人大代表建议、批评意见612件，办理政协委员提案1527件。城乡基层自治组织建设进一步强化，基层民主管理制度进一步健全，社会治安综合治理进一步加强。

 精神文明建设成效显著。广泛开展形式多样、内容丰富的精神文明创建活动，人民群众的文明素质和社会文明程度进一步提高。

 政府自身建设得到加强。引深学习实践科学发展观活动，大力开展“三责”教育。不断转变政府职能，行政效能明显提高。各级政府制度建设与应急管理体系日臻完善。煤焦领域反腐败工作和工程建设领域突出问题专项治理扎实开展。监察、审计等工作卓有成效。

 各位代表！发展成就令人振奋，发展历程令人难忘。

五年来，我市先后荣获全国双拥模范城、义务教育均衡发展先进地区、农村公路建设示范工程先进单位、社会治安综合治理“长安杯”，省和谐社会建设先进市、节能环保优秀奖、新农村建设先进市、安全生产先进单位、省级卫生城市等荣誉称号。经过五年的发展，晋中站在了一个新的历史起点上！

 五年来，我们举全市之力，圆满举办了奥运火炬平遥传递活动，大力支援南方抗击雨雪冰冻灾害，扎实开展汶川特大地震救援工作，全力防范甲型H1N1流感疫情，以经济社会发展和安保工作的优异成绩，共同度过了共和国六十华诞！五年来，我们面对复杂多变的发展形势和环境，从容应对来自各方面的挑战和困难，各级政府科学判断形势、驾驭市场经济、应对复杂局面、依法执政和总揽全局的能力不断增强，广大公务员服务意识、服务质量和服务效率显著提高，全市人民同心同德、和衷共济，形成了科学发展、赶超跨越的强大气势！

 特别是2009年，面对国际金融危机带来的困难和挑战，我们坚定信心、奋力自救，深入落实“三保”措施，推动了全市经济社会平稳较快发展。我们抓资金、抓市场，月调度、季分析，创环境、强服务，以空前的力度支持帮助企业度难关；我们主动出击，赢得了国家和省更多的资金支持，实施了一批重大项目，积蓄了发展后劲；我们抢抓市场机遇，利用倒逼机制，痛下决心调结构，抓住了新一轮经济增长的先机；我们迎难而上，高效运作，在城乡基础设施建设上取得了重大突破，解决了一批事关群众切身利益的突出问题。我市在金融危机中逆势而上，书写了本届政府凝心聚力、众志成城的浓重一笔！

 过去五年的成就和进步，来之不易。这是我们认真贯彻中央和省重大决策的结果，是省委、省政府和市委正确领导的结果，是市人大、市政协有效监督、大力支持的结果，是全市人民团结奋斗的结果。在此，我代表市政府，向全市工人、农民、知识分子、各级干部和离退休老同志，向全体人大代表、政协委员、各民主党派、工商联、人民团体、无党派人士，向关心帮助晋中发展的中央、省驻地单位和驻地部队、武警官兵，向积极参与晋中建设的海内外朋友，表示衷心的感谢！

 五年的丰富实践，我们积累了许多宝贵经验。我们始终坚持科学发展观，勇于用“跨越赶超”凝聚人心、激励斗志，营造快人一步、争先争上的发展之势；我们始终坚持解放思想，敏于乘势造势、创造机遇，通过开放提速，不断激发又好又快发展的生机与活力；我们始终坚持提高发展质量和效益，善于在统筹发展中重点突破，依靠项目带动，加快经济结构调整和发展方式转变；我们始终坚持创优发展环境，敢于拿出过硬招数破解发展难题，消除一切不利因素，全面增强吸引力、竞争力；我们始终坚持把广大人民群众的利益放在首位，致力于构建和谐社会，坚持执政为民、依法行政，让改革发展成果惠及城乡居民!

 我们也清醒地认识到，我市欠发达的基本市情没有改变，经济社会发展特别是政府工作还存在不小的差距。一是经济运行中一些突出问题和深层次矛盾依然存在，产业结构不合理、资源环境代价大、城乡发展不平衡等方面还表现明显；二是影响经济发展的体制机制障碍还不少，改革创新的任务依然繁重；三是涉及城乡居民切身利益的劳动就业、社会保障、教育卫生、收入分配、安全生产等问题，有待进一步解决；四是社会管理和公共服务比较薄弱，政府自身建设与形势的要求和人民的期望还有很大的距离。我们一定要保持清醒头脑，以对党和人民高度负责的使命感和紧迫感，更加兢兢业业、扎扎实实地做好各项工作，不遗余力地把晋中改革发展大业推向前进！

二、对下届政府工作的建议

 今后五年，新一届政府肩负着圆满完成“十一五”目标、奋力推进“十二五”规划、全面建设小康社会的历史重任。

 在前进的道路上，我市发展面临着难得机遇。全球经济正在摆脱国际金融危机影响，“后危机时代”新一轮技术和产业革命即将兴起，为我们扩大参与国际国内产业分工、加速特色产业发展创造了条件。全国经济回升向好，中部崛起战略深入实施，经济要素跨区域流动更加自由和广泛，有利于我们承接产业转移、加快区域一体化发展。我省争取成为“国家资源经济转型发展综合配套改革试验区”，十大产业调整振兴规划等重大政策启动实施，极大提升了转型发展的政策优势，对于促进我市经济结构调整和发展方式转变具有重大意义。太原都市圈建设逐步发力，我市借助省城资金、技术、人才、信息和市场资源，加快经济社会互动发展、推动城镇化进程，成为现实选择。全市人民谋发展、促赶超的心愿和期盼更加强烈、动力日益增强、活力不断迸发，我市进入了发愤图强、赶超跨越的全新阶段。只要我们牢牢把握机遇，振奋精神，不甘人后，埋头苦干，负重拼搏，一定能够创造新的成就，谱写新的篇章！

 下届政府工作的基本思路是：高举中国特色社会主义伟大旗帜，以邓小平理论和“三个代表”重要思想为指导，深入贯彻落实科学发展观，围绕“建设山西中部最具活力经济带和城市群”目标，以实施经济转型、赶超发展“两大战略”为重点，以新型工业化、特色城镇化、农业现代化为主线，以项目带动、科教领先、改革开放、区域融合、民生改善、环境创优“六大工程”为抓手，努力把晋中建设成为“转型发展先导区、晋商文化复兴地、现代宜居生态市”。

 努力实现六大目标：

 经济总量翻番，实力更强。全市地区生产总值、规模以上工业增加值、固定资产投资、财政总收入五年增长一倍以上，力争“再造一个晋中”，综合实力达到全省上游水平。

 发展方式转变，结构更优。构建资源节约、环境友好的绿色经济体系，煤焦等传统产业比重明显下降，装备制造、新型材料、现代农业、现代物流、文化旅游迅速发展，高新技术产业全面起步，产业结构、布局结构、投资结构更趋合理，民营经济、循环经济、园区经济比重大幅提升。

 生态文明优先，环境更好。构建蓝天碧水、低碳发展的环保生态体系，单位GDP综合能耗继续降低，主要污染物排放总量大幅减少，森林覆盖率、林木绿化率逐年提高。

 宜居建设加快，城乡更美。构建宜居宜业、统筹协调的城乡发展体系，“四城联创”目标基本实现，城市功能日益完善，新农村建设成效明显，城镇化率达到50%以上。

 改革开放深入，活力更足。构建又好又快、充满活力的新型体制机制，科技创新能力增强，人才智力快速成长，重大改革取得突破，开放程度大幅提高，直接利用外资和进出口总额保持较快增长。 社会和谐进步，人民更富。构建全面发展、公平正义的和谐社会体系，城乡就业更加充分，保障体系更加健全，各项社会事业全面发展，城乡居民收入大幅增加，人民生活一年更比一年好！

 根据以上基本思路和奋斗目标，应把握好六项原则：

 坚持整体推进和重点突破并重。以“争为三晋先”为发展理念和工作标准，统筹推进经济、社会、生态协调发展，在绿色转型、文化强市、城乡统筹等重点领域，改革探索、先行先试、扩大优势、彰显特色，通过点上突破带动面的提升。

 坚持集聚发展和融合发展并重。科学配置资源，引导产业集聚，壮大产业集群。主动融入太原都市圈，一体规划、同城建设、错位发展。

 坚持城市经济和县域经济并重。坚持两手抓，强化城市经济主导，夯实县域经济基础，实现城市经济与县域经济互动发展。

 坚持优进劣退和放手发展并重。大力淘汰落后产能，有序推进资源整合和产业重组，提高优势产业竞争力。放手发展民营经济，引导民间资本投向法律法规未明确禁止准入的领域。

 坚持科技创新和环境创优并重。转变不适应不符合科学发展观的思想观念，增强城市持续创新能力，打造一流的发展环境，努力形成鼓励创新、创造、创业的社会氛围。

坚持全民共建和全民共享并重。动员全市力量，共建和谐社会，强化人本理念，秉持公平正义，推进基本公共服务均等化，让广大人民群众过上幸福有尊严的生活。

 各位代表！未来五年的发展充满希望，新一届政府使命神圣、责任重大。我们坚信，有市委的正确领导，有市人大、市政协的监督支持，有历届市政府打下的良好基础，尤其是有310万勤劳智慧的晋中人民的共同努力，一个经济更加发展、民主更加健全、科教更加进步、文化更加繁荣、社会更加和谐、人民生活更加殷实的新晋中，一定会在三晋大地崛起!

 三、2010年工作安排

 各位代表！2010年，是全面完成“十一五”战略部署、谋划布局“十二五”经济社会发展的重要一年，是新一届政府带领全市人民谱写美好生活新篇章的起始之年。做好今年的各项工作，意义十分重大。

 今年的主要预期目标是：地区生产总值增长11%；规模以上工业增加值增长12%；全社会固定资产投资增长12%；财政总收入增长13.91%，一般预算收入增长5.05%；外贸进出口总额增长20%；社会消费品零售总额增长17%；城镇居民人均可支配收入增长10%；农民人均纯收入增长8%。

 约束性指标是：万元生产总值综合能耗下降3.87%；二氧化硫排放量下降2%，化学需氧量控制在2.19万吨以内；居民消费价格指数涨幅控制在3%左右；城镇新增就业人数3.2万人以上，城镇登记失业率控制在4%以内，人口自然增长率控制在6‰以内。

 按照以上目标，在积极做好“十二五”经济社会发展规划编制工作的同时，重点抓好十方面工作：

（一）坚持多措并举，全力保持经济平稳较快增长。

 继续加大项目投资力度。切实抓好产业开发和基础设施投资，确保全市固定资产投资完成440亿元以上。一是积极扩大产业投资规模。全市538项重点工程力争完成投资405亿元，其中市级72项重点工程力争完成投资190亿元。突出落实好东方希望集团循环产业园区、北六堡铁路物流中心和美特好经纬农副产品配送中心等一批重大项目。二是继续强化基础设施建设。加快松塔水电站、石膏山水库、恋思水库和泽城西安水电站二期项目建设进度，做好引黄南扩晋中供水工程前期工作。加快左权220千伏变电站等输变电工程建设。推进大西客专、太中银、和邢等铁路建设，抓好汾邢、龙城、平大3条高速公路建设和130公里二级公路改造，新增公路里程215公里。三是大规模开展招商引资。力争引进项目65个、资金110亿元。四是做好项目服务和管理。完善领导包项目制度，建立土地、资金、环境等要素“打包支持”机制。五是加强项目库建设。实施动态管理，提高储备质量。

 积极扩大消费需求。大力发展连锁经营、物流配送、电子商务等新型消费业态。切实抓好放心早餐工程试点和推广工作。完成“万村千乡市场工程”门店200个、“新网工程”门店100个。做好家电、汽车、摩托车下乡和以旧换新工作。

 努力扩大对外贸易。建立政府服务机制，培育机电、农副产品等新兴出口产品。完善重点企业出口退税绿色通道，支持企业尤其是民营企业办理对外贸易经营者备案登记。加大玻璃器皿等行业整合重组和设备更新改造力度，引导企业调整产品结构，开拓新兴市场。努力扩大进口，引进一批先进技术装备。

 切实做好帮扶企业工作。引深“三个一”帮扶活动，落实好“五缓四降一补”等措施，帮助企业解决生产经营中的困难。规模以上工业企业实现利税、利润力争分别增长15%和12%，亏损企业亏损额下降40%。继续坚持月分析制度，建立健全监控预警体系，加强经济运行监测。认真落实省“百强潜力企业培育工程”，引导企业跨县市、跨行业联合、兼并、重组，积极培育一批大公司、大集团。

加快县域经济发展。支持东山地区发展以煤电化为主的循环经济，壮大平川地区装备制造、纺机、液压、玻璃器皿、玛钢、食品医药等产业集群。建立健全县域经济考核体系，不断完善分组竞赛与单独考核相结合的考核办法，进一步激发县域经济发展活力，促进县域发展竞相赶超、争先进位。

 各位代表！今年的发展环境将好于去年，但面临的形势还很复杂。保持经济平稳较快发展，仍然是我们的首要任务。我们必须全面、正确判断形势，充分利用有利条件和积极因素，牢牢把握主动权，全力以赴保增长！

（二）加快转变发展方式，力争在调整优化经济结构上取得重大突破。

 引深煤炭资源整合、煤矿企业兼并重组。突出抓好主体做实、技术改造和关闭矿井工作，整体提升煤炭行业的装备、管理、安全生产水平和综合效益。完善24个主体企业法人治理结构，大力推进阳煤寺家庄、汾西柳泉、灵石灵北等大型现代化矿井建设，在严格程序、严格标准、确保安全的前提下，加快符合条件的煤矿复工复产，兼并重组后保留矿井力争全年原煤产量达到4000万吨。加强监管，防止已关闭矿井死灰复燃和私挖滥采现象发生。稳步推进非煤矿山企业资源整合。

 全面落实产业调整振兴规划。一是加快改造提升传统产业。焦炭产业要做好产能整合、企业重组、技术升级、布局优化工作，实施关小上大，力争焦化企业减少到25个、产能保持在2500万吨。电力产业要瞄准一流水平，重点发展热电联产和超超临界发电机组，加快左权大电厂、寿阳中电投电厂建设，确保山西瑞光、平遥耀光电厂投产，年内总装机容量达到320万千瓦。煤化工产业要坚持“煤焦化、煤电化、煤冶化”思路，推进“焦”“化”并行，杜绝有“焦”无“化”，加快介休益达、灵石聚源30万吨甲醇、昔阳40万吨电石、灵石4A沸石等项目建设。二是培育壮大新兴产业。机械装备产业要积极做好吉利轿车、中航工业专用车和一汽靖烨重卡发动机三大项目的引进和建设工作。食品医药产业要重点抓好果蔬、食醋、肉制品和绿色健康食品加工，加快广生植物胶囊、中远威溶栓片等项目建设，扩大德元堂红花黄色素注剂销售市场。三是加强工业园区建设。在规范提升市级主抓7大园区的同时，规划建设山西新能源汽车·机械装备制造区、液压元器件国家示范园区和纺机制造国家示范基地。

 提升服务业规模和素质。继续大力实施“3+7”发展战略。现代物流业要启动太原都市圈晋中综合物流园区规划与一期建设项目，加快山西能源工业国际分销物流港建设，力争三晋国际商贸物流城、山西新华图书物流中心竣工运营，山西粮食物流中心开工建设。积极发展包装、流通、加工、配送、信息处理等多种物流形态，形成“多功能区域性商贸物流中心”基本框架。旅游业要加快平遥古城和介休绵山国家5A级旅游区、祁县乔家大院等景区建设，积极发展生态休闲和农家乐旅游项目，不断完善游客中心、特色餐饮、星级饭店、工艺品、演艺节目等要素，创建国家旅游服务业标准化试点城市。全市旅游总收入力争过百亿。房地产业要实施好北部新城年度建设项目，启动高档商业、商务地产和特色住宅片区建设。

 大力发展循环经济、低碳经济和高新技术产业。做好全市循环经济发展总体规划编制，扩大介休市、东阳镇、中煤九鑫工业园区等省级循环经济试点成果，加快左权龙泉、灵石两渡等循环经济工业园建设。切实抓好低碳生产、技术产品开发推广和项目引进工作，力争在甲醇燃料发动机研发、节能环保新材料应用等方面取得突破。积极发展新能源、电子信息、生物产业、节能环保等高新技术产业，争取国家纺机检测中心落户晋中。

 加快民营经济发展。认真贯彻落实《国务院关于鼓励和引导民间投资健康发展的若干意见》，鼓励和引导民间资本进入基础产业和基础设施、市政公用事业、社会事业、金融服务等行业和领域，突出抓好煤焦领域退出资本就地转化工作。加强对中小企业、民营企业生产经营运行监测分析。加快建设公共服务平台、信息服务网络和中小企业创业基地，促进民营企业转产转型。

 各位代表！我市作为资源型欠发达地区，调整经济结构、转变发展方式刻不容缓。我们必须抓住难得机遇，大胆创新、有所作为，加快推动我市经济发展进入创新驱动、内生增长的轨道！

(三)强化规划、建设和管理，加快建设现代宜居生态城市。

 做实规划工作。重点抓好城市总体规划、北部新城大学园区用地控制性详细规划等9个项目。组织开展控制性详细规划编制，全市控规覆盖率提高10个百分点，介休、寿阳力争实现控规全覆盖。完成县域村镇体系、建制镇、历史文化名镇名村、国省级重点镇和新农村规划编制工作。扎实做好县、乡土地利用总体规划修编。

 切实加强市政重点工程建设。抓好市城区5大类55项重点工程建设，总投资27.4亿元。推进热电联供和城市排水两大管网配套工程，力争热电联供新入网540万平方米。实施中都路拓宽改造。加快潇河生态公园、晋商公园建设。

 全面改善城市管理和服务。继续深入开展“四城联创”活动。引深城乡清洁工程，以精细化管理、长效机制建立为重点，狠抓环境治理，对违法建设、建筑工地、便道街巷、环境卫生进行集中专项整治。坚持民生优先，注重功能提升，加强市政公用行业的运营管理，确保设施运行安全稳定，公共服务持续增强。大力发展公共交通。高标准做好“治超”工作。

 各位代表！今年是晋中建市十周年。我们的城市还很年轻，城市发展的路还很长。只要全市人民齐心协力，攻坚克难，就一定能够把我们的城市建成现代、宜居、生态的美丽家园！

（四）统筹城乡发展，全面提高“三农”工作水平。

 改善农业基础条件。实施最严格的耕地保护制度，稳定粮食生产，确保粮食安全。继续开展水利基础设施建设年活动，规划和实施100处重点水利工程，新建改善节水耕地面积10万亩，改造中低产田4万亩。实施农业机械化推进工程，新建农机大院30个，新发展农机大户50个。加大农业科技投入，大力引进和推广新技术、新品种。加快推进农业信息化，规范300个“农村信息服务超市”。

 大力发展现代特色农业。围绕创建国家级现代农业示范区目标，加快“晋中现代农业示范区”建设，抓好20个核心项目。推进二轮优势农产品区域布局和省级优势农产品基地县建设。新建和改扩建100个养殖小区（园区），培育50个现代畜牧企业，确保农民人均畜牧业收入增长8%。探索建立蔬菜发展风险基金和商业保险，鼓励土地经营权向设施蔬菜经营户流转，新发展设施蔬菜4万亩，力争提供农民人均纯收入600元以上。扩大丘陵山区核桃标准化栽培，强化平川红枣综合管理，新发展干果林10万亩，力争提供农民人均纯收入300元以上。推进农业产业化经营。抓住省实施“513”工程契机，突出抓好15个亿元龙头企业、10个转型加工企业和25个潜力型加工企业，新上农产品加工技改扩建项目20个。新规范农民专业合作社1000个，农民入社率达到15%。实施百万农民大培训“六个一”工程。完善农产品质量安全体系，做好“三品”认证工作。

 扎实推进新农村建设。推动城市基础设施向农村延伸，促进城乡共建共享。强化农村交通、医疗卫生、教育、商贸、通讯信息、体育文化设施建设，加快旧村改造步伐。全面完成“五个全覆盖”工程任务。促进现有500个“十有十个一”达标村升级上档。实施开发扶贫规划，完成83个整村推进项目任务。推进“雨露计划”，培训农村富余劳动力2000名，转移率达到70%以上。

 加大农村改革力度。继续深化农村土地经营制度改革，健全土地流转机制，推广祁县西六支专业合作社经验，推进农业适度规模经营。全面推开集体林权制度改革，70%以上村完成主体改革任务。扎实推进基层农技推广体系改革。加快城乡水务一体化改革和水工程供水价格改革。统筹开展支农资金管理、农村金融体制等改革。

 加快城镇化进程。抓好城镇公共基础设施和公共服务设施建设，每县选择2到3个中心镇，培育发展特色小城镇。深化就业、社保、医疗等方面改革，逐步实现农民工在劳动报酬、子女上学、公共卫生、住房租购以及社会保障等方面与城镇居民享有同等待遇。重点推进户籍制度改革，放宽城市落户条件，使在城镇稳定就业和居住的农民有序转变为城镇居民。

 各位代表！“三农”问题事关全市发展大局，事关广大农民生计，是我们的感情所系。让农村更加富裕、繁荣、和谐，我们责无旁贷！

（五）深入推进改革开放，为经济发展注入新的动力和活力。

 实施国企改革攻坚。如期终结晋华、锦纶、轻机3户政策性破产企业的法律程序，处理好有关遗留问题，做好善后工作。启动晋中药材公司、格芙兰公司、晋中建设集团改制工作。积极推进市直部门和县属国有企业改革，鼓励重点企业实施并购，壮大规模。继续推进政府社会公共管理职能与国有资产出资人职能分开，进一步健全完善国有资产监督管理体系，促进国有资产保值增值。

 扎实推进行政管理体制改革。按照省委、省政府要求，完成市县政府机构改革、市县事业单位清理和分类改革任务。继续推进医药卫生体制改革，60%的城市社区卫生机构和乡镇卫生院建立基本药物制度。加快文化体制改革，积极推进艺术表演团体转企改制和文化事业单位改革。加快供销社体制改革。

深化财政体制改革。完善财政监督体系，建立健全覆盖所有政府性资金和财政运行全过程的监督机制。强化对预算编制、执行、调整的管理和约束，提升政府理财水平。巩固和深化“小金库”治理成果，构建长效机制。优化完善财政分配体制，建立财权与事权相匹配、有利于区域经济发展的财政管理体制。

 大力推进对外开放。进一步增强对外开放意识，加强对外交流合作，做好上海世博会、南昌中博会、第三届能源博览会参展工作。创新外经外贸工作，主动与大公司、大集团对接，促成一批大项目落户晋中。实施“走出去”战略，鼓励能源、化工、纺机、液压等优势企业组建国际化生产体系和营销网络，拓展企业发展空间。充分发挥人力资源优势，大力发展以工程承包为主的劳务输出。

 加快区域一体化发展。抓住中部崛起战略和太原都市圈建设机遇，加强区域合作，促进与省城的对接互动。以资源资本资金为纽带，以企业为主体，加快与周边地区的产业融合和企业联合。重点在新能源汽车、机械制造、大型物流、农副产品批发、房地产业以及道路体系等方面取得重大合作成效。

 各位代表！我们一定要围绕解决阻碍经济社会发展的深层次矛盾和问题，坚定不移地深化改革、扩大开放，奋力攻坚、务求突破！

（六）加快节能减排和生态建设，促进经济社会可持续发展。

 加大节能力度。引深“双百”企业节能行动计划，开展六大高耗能行业能效对标，加强新上项目能耗评估审查，加大节能监察执法力度，引导节能服务产业发展。实施能源合同管理，加大节能项目实施，对煤焦、玛钢、玻璃器皿、碳素工业燃煤炉窑、电机进行节能改造和跟踪管理，全年淘汰落后产能20万吨和13.9万千瓦。积极推进节约型机关建设。

 狠抓污染减排。实施重点污染企业强制性清洁生产审核和减排项目分类处置，加大燃煤电厂二氧化硫削减治理力度。强化重点污染源在线监测监控。加强城市污水处理厂运行监管。市城区垃圾无害化处理率达到60%以上，县（区、市）垃圾处理厂全部竣工并投入使用。境内河流断面水质全面达标。市城区空气质量稳定达到国家二级标准。

 大力开展造林绿化。推进省级林业生态市建设，实施碳汇合作造林，加强矿山和汾河流域生态恢复与治理，造林绿化35万亩以上，植树1000万株以上。高标准组织好全省造林绿化现场会。

 各位代表！在节能减排和生态建设这个问题上，我们的决心不动摇，力度不减弱，无论面临多大困难，一定要打赢这场攻坚战!

（七）提高工作标准，不断创优发展环境。

 提升政务环境建设水平。继续做好精简审批和放权工作。深化行政审批制度改革，大幅度清理部门条规、清算收费项目、清查审批事项，切实把审批减到位、环节压到位、收费降到位。健全完善四级政务服务网络，加快电子政务平台建设，推行网上行政审批和电子行政监察。拓展“12345”便民热线服务范围，落实百件实事网上办工作，不断完善宁静生产日和重大项目全程代理服务制。

 在金融生态环境建设上取得突破。加快发展资本市场，鼓励商业性小额贷款组织增资扩股。继续引进银行、证券、保险等金融机构设立分支机构或办事处。强化要素市场建设，大力培育和发展会计师事务所、律师事务所及产权交易、资产评估、证券投资咨询和信用评级等中介机构。积极扶持辰兴集团上市。完善担保体系，加大县级担保公司资本金注入，鼓励商业性担保机构发展。探索债券、票据、信托等融资渠道，鼓励符合条件的企业发行短期融资券和中期票据，争取发行企业债券8.5亿元。拓展商业银行县级分支机构，发展村镇银行、农村资金互助组织、农村合作银行等金融试点。加强财政融资平台建设。

 推进人才成长环境建设。积极创造条件，吸引一批高层次创业人才和创新团队来我市创业发展。进一步充实完善优秀人才引得来、留得下、用得好的政策与办法，加快构建有利经济发展、具有地方特色的引才机制和育才机制。突出抓好项目建设所需人才的组织、培训和引进。

 各位代表！抓发展必须抓环境，抓环境就是促发展。我们一定要勇于自我革命，敢于争创一流，全力把我市打造成发展的高地、投资的洼地、人才的集聚地！

（八）全面发展社会事业，切实保障和改善民生。

 扎实做好城乡就业。继续推行“九个一批”就业模式，以高校毕业生、登记失业人员和农民工为重点，强化职业技能培训、“一对一”就业援助、公益性岗位“托底”安置、创业就业等措施，完成劳动力职业技能培训8.2万人，城镇新增就业3.2万人，让广大劳动群众实现体面劳动。

 进一步完善社保体系。完善各类保险制度，扩大保险覆盖面，提高保障标准。推进廉租房建设，加大棚户区和农村危房改造力度。落实好城乡低保、农村“五保”、社会救助等措施，大力发展社会福利和慈善事业，加快残疾人社会保障体系和服务体系建设。

 全面发展社会事业。推进义务教育均衡发展。义务教育学校调整到1000所左右；普通高中学校调整到30所，高中阶段教育普及率达到92.3%以上；建设好20所职业中学，促进普通高中和职业教育、成人教育协调发展；切实加强校园安全保卫工作。大力发展医疗卫生事业。健全疾病预防控制长效机制，提高应对突发公共卫生事件的能力；全面完成村卫生室建设任务，提高县乡村医疗卫生机构达标率；全面推开门诊住院双统筹工作，提高新农合筹资水平和最高支付限额。增强科技创新能力。继续实施“463科技支撑工程”，切实加强与中科院、本地院校等科研院所的产学研合作，发挥科技创新担保资金引导作用，加快企业工程中心、技术中心建设。大力发展公共体育事业。广泛开展全民健身运动，完成农民健身场地800个，农村体育场地实现全覆盖。继续稳定低生育水平，实施“优生促进工程”，提高人口素质。加大管理和保护力度，确保文物和档案安全。

 切实加强社会管理。强化基层民主管理，妥善处理好土地征用、企业改制等各类社会矛盾。完善自然灾害预警体系和突发事件应急处置机制，加强应急队伍和装备建设。深入开展“三责”教育，实施以安全生产和社会稳定监管为主要任务的网格化社会管理。

 持之以恒抓好安全生产。围绕本质安全目标，深入开展“安全生产基层基础建设年”活动。突出抓好煤炭安全生产，认真落实“双逐月”制度，强化安全巡查、“五人小组”驻矿监管等一系列措施。增加安全生产投入，实施煤矿安全监管信息化项目。大力推进煤矿劳动用工制度改革。加强道路交通、非煤矿山、危险化学品、建筑施工、森林防火、消防等行业和领域的安全生产，抓好重大疫病预防控制，加强地质灾害治理，坚决遏制重特大事故发生。实施食品药品放心工程。严格各级政府安全监管主体责任和企业安全生产主体责任，开展全员全岗安全培训和应知应会能力考核。加大责任追究和惩处力度，强化事前责任追究，严厉打击、依法追究非法违法生产经营行为。开展安全乡村（社区）创建活动。

 各位代表！幸福和谐是我们的美好追求，解决好事关群众利益的突出问题，是人民政府义不容辞的责任。我们要尽最大的努力，让广大人民群众的生活越来越好、更加幸福！

（九）加强民主法制和精神文明建设，促进和保障经济社会又好又快发展。

 加强民主法制建设。扎实推进依法治市进程。认真执行市人大及其常委会的决议决定，自觉接受人大及其常委会的法律监督、工作监督和政协的民主监督，主动接受新闻舆论和社会公众监督，认真办理人大代表、政协委员的议案、提案和建议。广泛听取各民主党派、工商联、无党派人士的意见和建议。加强基层民主建设，健全政务公开、厂务公开、村务公开等制度，保障人民群众的知情权、参与权、表达权、监督权。支持工会、共青团、妇联、科协等群团组织的工作，支持法院、检察院依法独立行使审判权、检察权。深入推进“五五普法”活动，继续引深“法律六进”活动。搞好法律援助工作，维护妇女、儿童、残疾人和社会弱势群体的合法权益。加强社会治安综合治理，严厉打击各类刑事犯罪活动，创建平安晋中。

 加强社会主义精神文明建设。大力弘扬以爱国主义为核心的民族精神和以改革创新为核心的时代精神，认真挖掘“晋商精神”的积极内涵，鼓舞全市人民保持昂扬向上的精神状态。加强社会公德、职业道德、家庭美德、个人品德建设。深入开展文明和谐创建活动，培育文明新风，促进社会和谐。

 全力推进“文化晋中”建设。实施《文化产业发展规划纲要》，突出文博会展、影视拍摄基地、工艺美术品、演艺等特色，积极发展休闲娱乐文化、晋商文化、民间文化、红色旅游文化产业，加快国家级晋中文化生态保护区建设，抓好绵山清明（寒食）博物馆、榆次影视拍摄基地、市级文化基础设施建设等项目，重点扶持20个文化企业建设。加快发展文化事业，建立健全公共文化服务体系，推进农村“2131”电影放映和“农家书屋”建设工程。发展新闻出版、广播影视、文学艺术事业。

 各位代表！晋商精神是我们的宝贵财富。作为晋商文化的发源地和主要传承地，在社会主义市场经济条件下，我们一定要承担起复兴晋商文化的历史使命！

（十）切实加强政府自身建设,努力建设人民满意的服务型政府。

 建设学习型政府,提升创新力。坚持和创新政府班子集体学习和工作交流制度，积极采用专家讲座、形势报告、专题讨论等多种形式，系统学习经济社会发展和政务管理知识。坚持理论联系实际，学以致用、用以促学，不断提高谋划发展、统筹发展、创新发展的本领。

 建设法治政府，增强公信力。加强政府法制工作，认真落实《全面推进依法行政实施纲要》，按照决策、执行、监督相协调的原则，建立健全民主科学决策的体制机制。严格行政执法责任制、过错责任追究制和执法督查制等制度，提高依法行政水平，让权力在阳光下运行。

 建设效能政府，提高执行力。落实“争为三晋先”要求，每个部门、每个环节、每项工作都要当先锋、争上游、创一流。探索建立科学统一的政府绩效考核指标体系，逐步形成与政府管理创新相适应的激励约束机制和社会化评价体系。自觉维护市委决策的严肃性，完善重大事项和重点工作跟踪落实机制。

 建设廉洁政府，强化约束力。全面推进惩治和预防腐败体系建设，落实党风廉政建设责任制，努力形成靠制度管人、制权、理事的有效机制。继续深化煤焦领域反腐败专项斗争，深入开展工程建设领域突出问题专项治理。加强公务员教育、管理和监督，建设勤政廉洁的公务员队伍。强化审计监督、行政监察。厉行节约、勤俭办事。

 各位代表！我们所有的努力，所做的一切，就是要为人民服务、对人民负责、受人民监督！

 今年，市政府要全力办好以下十件实事：一是完成130万平方米中小学校舍安全改造加固任务。二是积极扩大新农保试点，对2459个就业困难人员实施就业援助。三是新建、改扩建12所农村敬老院。四是对150名贫困残疾儿童实施康复救助。五是新开工建设保障性住房10570套、82.57万平方米，完成5000人扶贫移民任务，确保全面完成国有重点煤矿棚户区改造任务。六是解决好35万农村人口的安全饮水问题。七是完成600公里具备条件建制村新建水泥(油)路任务。八是新建300家连锁农家店，建设改造14家放心粮油配送中心，改造提升90家示范中心店，建设完善1500个经销网点。九是新（改）建829个村级组织活动场所。十是改造50条城市小街巷，其中市城区改造14条。

 各位代表！站在历史新起点，面对人民新期待，我们深感使命光荣，责任重大。让我们在省委、省政府和市委的坚强领导下，满怀发展激情，保持昂扬斗志，开拓创新，埋头苦干，加快转型发展先导区、晋商文化复兴地、现代宜居生态市建设步伐，为早日实现山西中部最具活力经济带和城市群目标而努力奋斗！
